# Burden of ovarian cancer in China from 1990 to 2030: A systematic analysis and comparison with the global level

**DOI:** 10.3389/fpubh.2023.1136596

**Published:** 2023-02-13

**Authors:** Ying Wang, Zhi Wang, Zihui Zhang, Haoyu Wang, Jiaxin Peng, Li Hong

**Affiliations:** Department of Obstetrics and Gynecology, Renmin Hospital of Wuhan University, Wuhan, China

**Keywords:** ovarian cancer, disease burden, epidemiology, risk factors, age-period-cohort analysis, joinpoint regression, prediction

## Abstract

**Introduction:**

Ovarian cancer (OC) is one of the major diseases threatening women's health and life. Estimating the burden trends and risk factors of OC can help develop effective management and prevention measures. However, there is a lack of comprehensive analysis concerning the burden and risk factors of OC in China. In this study, we aimed to assess and predict the burden trends of OC in China from 1990 to 2030, and make a comparison with the global level.

**Methods:**

We extracted prevalence, incidence, mortality, disability-adjusted life years (DALYs), years of life lost (YLLs), and years lived with disability (YLDs) data from the Global Burden of Disease Study 2019 (GBD 2019) and characterized OC burden in China by year and age. OC epidemiological characteristics were interpreted by conducting joinpoint and Bayesian age-period-cohort analysis. We also described risk factors, and predicted OC burden from 2019 to 2030 using Bayesian age-period-cohort model.

**Results:**

In China, there were about 196,000 cases, 45,000 new cases and 29,000 deaths owing to OC in 2019. The age-standardized rates (ASRs) of prevalence, incidence and mortality have increased by 105.98%, 79.19%, and 58.93% respectively by 1990. In the next decade, OC burden in China will continue to rise with a higher rate than the global level. The OC burden in women under 20 years of age is slowing down, while the burden in women over 40 years of age is getting more severe, especially in postmenopausal and older women. High fasting plasma glucose is the major factor contributing the most to OC burden in China, and high body-mass index has surpassed occupational exposure to asbestos to be the second risk factor. OC burden from 2016 to 2019 in China has increased faster than ever before, indicates an urgent need to develop effective interventions.

**Conclusion:**

The burden of OC in China has shown an obvious upward trend in the past 30 years, and the increase rate accelerated significantly in recent 5 years. In the next decade, OC burden in China will continue to rise with a higher rate than the global level. Popularizing screening methods, optimizing the quality of clinical diagnosis and treatment, and promoting healthy lifestyle are critical measures to improve this problem.

## 1. Introduction

OC is the most lethal malignancy of female reproductive system with poor prognosis. Unfortunately, 70% of OC patients are found to be in the advanced stage, and the 5-year survival rate is only 47.4% (1). High recurrence rate and chemotherapy resistance rate are the main reasons for the low survival rate. According to the latest data, OC ranks the 8th in incidence and mortality rate among women cancer in high-income areas, while it ranks the third among women in low- and middle-income areas, only after breast cancer and cervical cancer (2). China is a middle-income country with a population of 1.4 billion. It's reported that OC has replaced uterine cancer and became the second leading cause of death in gynecological cancer in China (3).

Many factors, such as population aging, economic development, lifestyle changes, and medical condition improvement, can affect the evaluation of OC burden. Therefore, understanding the current situation and future trends of OC burden is critical to develop appropriate prevention and control measures. Although there have been articles describing and assessing trends in the global burden of OC (4, 5), there are few comprehensive analyses of OC burden in China. Wang et al. has analyzed the disease burden trends of three major gynecological cancers including cervical cancer, endometrial cancer and OC in China, but only the mortality rates were analyzed (3). There is still a lack of rounded analysis including prevalence, incidence, mortality, DALYs, YLDs, YLLs and related risk factors of OC burden in China.

Using the data from GDB 2019, this study comprehensively described the temporal trend and age distribution of OC burden in China by discussing prevalence, incidence, mortality, DALYs, YLLs and YLDs from 1990 to 2019. We also used joinpoint regression method to figure out the trends in specific periods. Furthermore, we explored the effects of age, period and cohort factors on OC burden in China by using age-period-cohort analysis. Finally, we assessed the trends in attributable risk factors affecting OC mortality and DALYs, and projected trends in OC incidence and mortality over the next decade. This study has also compared the OC burden in China and the global level, which will provide more reference for the formulation and implementation of OC prevention and control policies in China.

## 2. Materials and methods

### 2.1. Data sources

Data in our study was obtained from GBD 2019 (http://ghdx.healthdata.org/gbd-results-tool). GBD 2019 contains comprehensive data of disease burden for 369 diseases and injuries with 87 risk factors in 204 territories and countries from 1990 to 2019. Data resources, disease classification, statistical models and measures to optimize data quality have been described previously in detail (6–8). Specifically, we downloaded the following data for subsequent analysis: (1) Age-specific data for OC on prevalence, incidence, deaths, DALYs, YLDs, and YLLs as absolute numbers, crude rates and ASRs including 95% confidence interval (95% CI), annually from 1990 to 2019 in China and the global level; (2) Age-specific population data from 1990 to 2019 and projected population data from 2020 to 2030 in China and the global level; (3) Associated risk factors attributable to OC from 1990 to 2019 in China and the global level.

### 2.2. Statistical analysis

#### 2.2.1. Descriptive analysis

We performed a descriptive analysis of both the temporal and age trends of OC burden in China compared with the global level. We also analyzed the temporal trends of risk factors contribute to OC from 1990 to 2019 by accessing population attributable proportion (PAF). All data used in descriptive analysis were prepared by using Microsoft Excel 2019, and statistical analysis were performed by using R software (version 4.2.2). A statistically significant *p* was <0.05.

#### 2.2.2. Joinpoint regression analysis

Joinpoint regression model was applied to evaluate temporal trend of OC burden in certain periods. This model has the advantage of avoiding the non-objectivity of typical trend analysis based on linear trends, as it estimates the changing pattern of age-adjusted rates by the least square method. In this model, the inflection points of the moving trend were figured out by summing up residual squares between the actual value and the estimated value using grid search method. The Monte Carlo permutation method was used to test whether the changes were significant. We determined the change trend of OC burden in certain periods by comparing annual percentage change (APC) which was calculated by two steps: (1) *y* = α + β x+ ε, in which y = ln (rate), x = year, β is the estimated value of the slope, and ε was the error term; (2) APC=100[exp(β) – 1]. Average annual percentage change (AAPC) was calculated to estimate overall OC burden trend from 1990 to 2019. APC > 0 means an increasing trend, while APC < 0 means a decreasing trend, and so does AAPC. This model was created by Joinpoint software (version 4.9.1.0).

#### 2.2.3. Age-period-cohort analysis

We used age-period-cohort model to explain the effects of age, period, and cohort on OC incidence and mortality. In this model, Net Drift reflects the overall time trend in disease rates which is similar to AAPC, the difference lies on that Net Drift takes into account both the effects attributed to period and cohort. Local drifts were used to estimate the average annual percentage changes of OC incidence and mortality rates for certain age groups. Age effect mainly means the effect of disease rate changes with age, and it is characterized by Longitudinal Age Curves which stands for the age-related natural course of the disease rate. Period effect reflects the influence of social, economic and cultural environment changes in different periods on the disease rate. Cohort effect refers to changes in disease rates due to different levels of exposure to risk factors among different generations. Relative risk (RR) was applied to evaluate the period and cohort effects. An intrinsic estimator based on Poisson distributions was conducted to access disease parameters in this model to overcome the multicollinearity problem between age, period, and cohort. To avoid overlapping information in adjacent queues, the age, period, and time interval of the queues must be equal. Thus, the ages were defined as 5–9, 10–14,…90–94, 95–100, and the incidence and mortality rates every 5 years were calculated. Age-period-cohort analysis was performed by Age Period Cohort Tool (http://analysistools.nci.nih.gov/apc/) (9).

Bayesian age-period-cohort (BAPC) model has been proved to have the highest coverage (with 95% CI) and it is well-fit for analyzing predictions of age-stratified cancer rates (10, 11). Based on age-specific population data from 1990 to 2019, projected population data from 2020 to 2030, and GBD world population age standard which is specified in Appendix Table 13 of a GBD 2019 (6), we applied BAPC model to forecast OC incidence and mortality rates for the next 10 years. The BAPC models were built from R packages INLA (www.r-inla.org) and BAPC (http://r-forge.r-project.org/).

## 3. Results

### 3.1. Temporal trends of OC burden in China from 1990 to 2019

In China, there were about 196,000 incident cases, 45,000 new cases for OC and 29,000 deaths due to OC in 2019. Compared to 1990, the crude rates of prevalence, incidence, and mortality in 2019 were all significantly increased by approximately 3 times. In addition, DALYs, YLDs, and YLLs of OC were 835,000, 25,000, and 810,000 years, respectively, and the crude rates of them in 2019 were 2.53, 3.14, and 2.51 times higher than those in 1990 (Supplementary Table 1).

In 2019, the age-standardized prevalence rate (ASPR), age-standardized incidence rate (ASIR), age-standardized mortality rate (ASMR), age-standardized DALYs rate (ASDR), and the ASRs of YLDs and YLLs of OC were 10.15, 2.29, 1.43, 40.53, 1.25, and 39.28 (per 100,000 population) in China, which have increased by ~105.98%, 79.19%, 58.93%, 47.88%, 91.78%, and 46.81%, respectively from 1990 to 2019, while the global burden of OC has remained roughly the same from 1990 to 2019 (Table 1).

**Table 1:** Change of age-standardized rates (per 100,000 population) in prevalence, incidence, mortality, DALYs, YLDs and YLLs for ovarian cancer between 1990 and 2019 in China and Global level.

**Measure**	**China**			**Global**		
	**ASRs (95% CI) in 1990**	**ASRs (95% CI) in 2019**	**Change of ASRs** **(95% CI)**	**ASRs (95% CI) in 1990**	**ASRs (95% CI) in 2019**	**Change of ASRs** **(95% CI)**
Prevalence	4.93 (3.84, 6.67)	10.15 (7.52, 12.99)	1.06 (0.27, 1.90)	12.72 (11.72, 14.38)	14.56 (12.85, 16.37)	0.14 (−0.03, 0.30)
Incidence	1.28 (1.00, 1.79)	2.29 (1.68, 2.88)	0.79 (0.08, 1.53)	3.42 (3.17, 3.84)	3.58 (3.17, 4.00)	0.04 (−0.11, 0.18)
Mortality	0.91 (0.70, 1.34)	1.43 (1.04, 1.81)	0.58 (−0.10, 1.28)	2.50 (2.32, 2.80)	2.43 (2.15, 2.66)	−0.03 (−0.16, 0.08)
DALYs	27.40 (21.18, 38.49)	40.53 (29.96, 51.58)	0.48 (−0.10, 1.13)	64.73 (59.29, 74.27)	64.34 (56.42, 71.52)	−0.01 (−0.16, 0.12)
YLDs	0.65 (0.42, 0.99)	1.25 (0.80, 1.76)	0.92 (0.15, 1.71)	1.72 (1.23, 2.22)	1.86 (1.34, 2.40)	0.08 (−0.08, 0.23)
YLLs	26.75 (20.59, 37.66)	39.28 (28.98, 50.34)	0.47 (−0.11, 1.13)	63.01 (57.52, 72.45)	62.49 (54.96, 69.27)	−0.01 (−0.16, 0.11)

DALYs, disability-adjusted life-years; YLDs, years lived with disability; YLLs, years of life lost; Change, indicators in 2019 minus those in 1990 by 1990; ASRs: age-standardized rates; 95% CI: 95% confidence interval.

**Figure 1:**
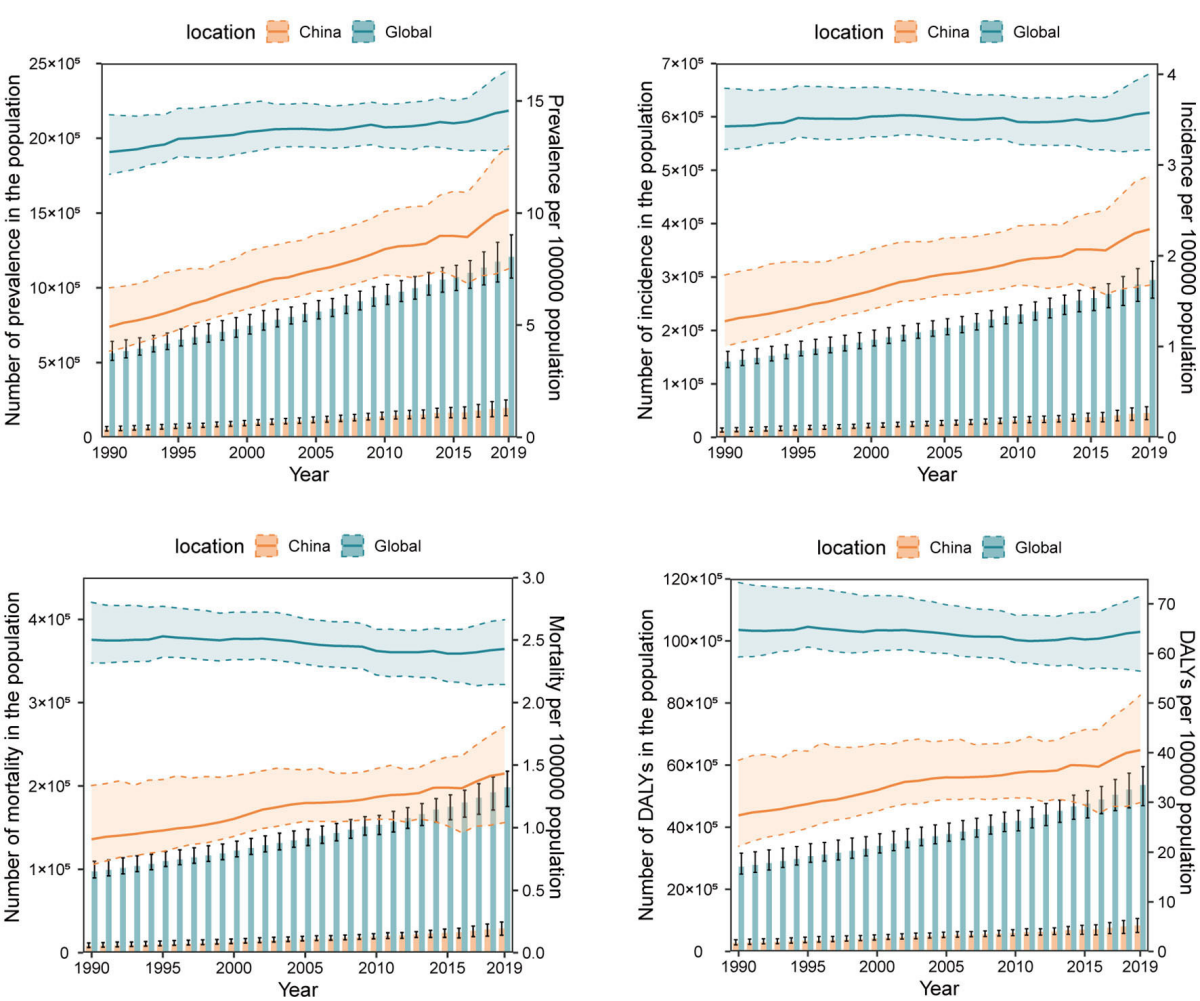
The numbers and age-standardized rates (per 100,000 population) of ovarian cancer prevalence, incidence, mortality, and disability-adjusted life-years (DALYs) from 1990 to 2019 in China and the global level. The bar chart represents numbers and the broken line chart represents age-standardized rates.

We also compared the trend of OC burden as numbers and ASRs in China and the global level year by year. Generally, the numbers of prevalence, incidence, mortality, DALYs, YLLs, and YLDs of OC in both China and the global level kept on increasing. The difference lies on that, while the ASRs of all these burden indicators in the global level generally hold steady from 1990 to 2019, the ASRs in China have risen apparently. It's noting that OC burden in China has ever remained stable from 2014 to 2016, but after that, the ASRs of all the burden indicators in China continued to increase at a faster pace than ever before (Figure 1, Supplementary Figure 1). Therefore, though the current ASRs of these disease burden indicators in China were still lower than those in the global level, they are likely to exceed the global level in the future if no measures are taken.

### 3.2. Burden trends of OC in different age groups in China from 1990 to 2019

**Figure 2:**
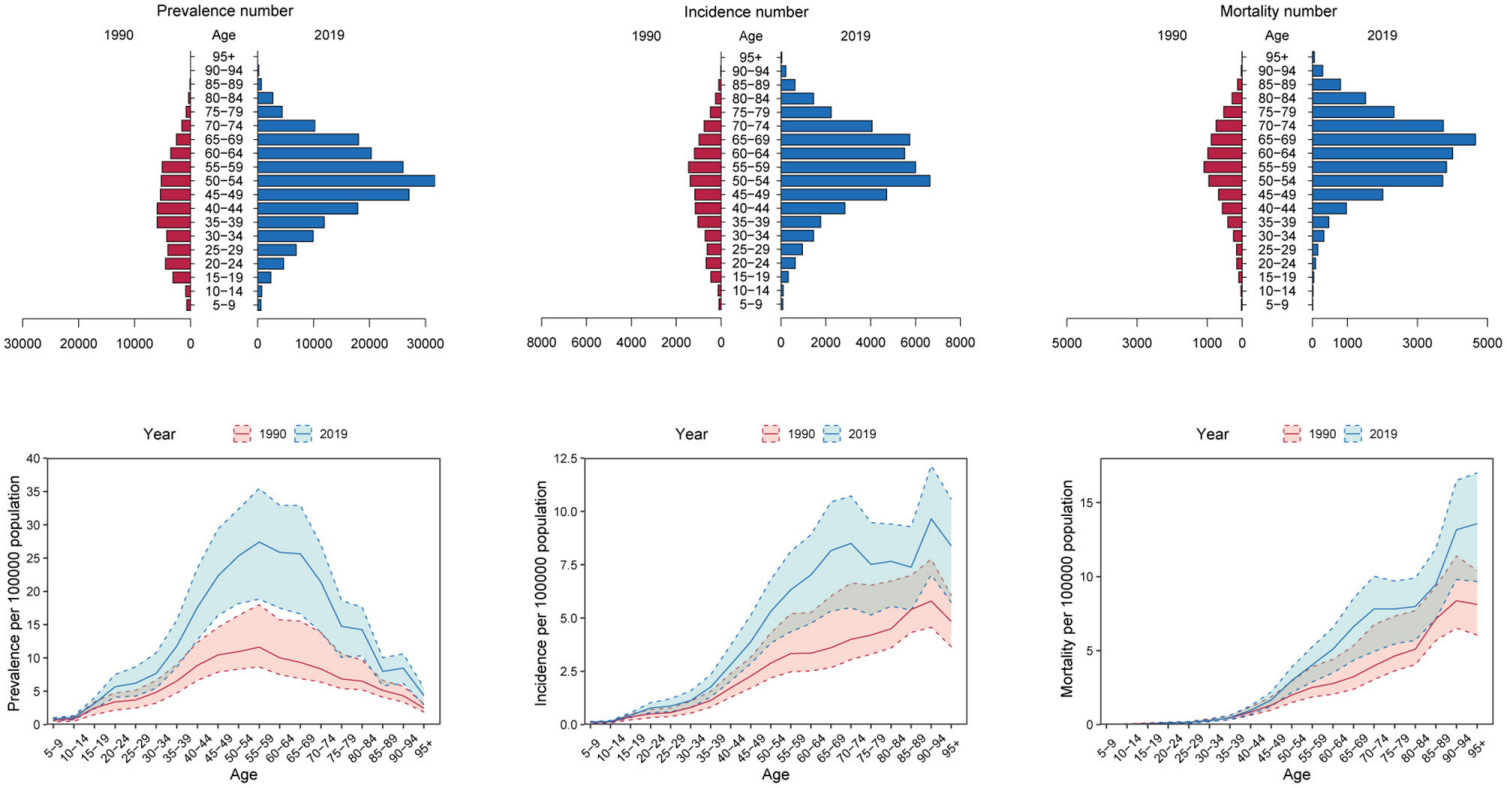
The numbers and crude rates of ovarian cancer prevalence, incidence and mortality in 2019 compared with 1990 in China.

In 2019, the numbers of prevalence, incidence, mortality, DALYs, YLDs and YLLs of OC in most age groups especially those over 40 years of age increased significantly compared to 1990, except for those under the age of 20 of whom the indicators remained stable. The numbers of prevalence, incidence, DALYs, YLLs, and YLDs all peaked between 50 and 54 years of age [Prevalence number: 31,690 (95% CI: 40,480, 22,730); incidence number: 6,650 (95% CI: 4,780, 8,520); DALYs number: 142,120 (95% CI: 104,370, 184,600); YLDs number: 4,080 (95% CI: 2,580, 5,930); YLLs number: 138,040 (95% CI: 101,150, 180,110)], while the numbers of mortality peaked between 65 and 69 years of age [4,660 (95% CI: 3,060, 6,010)] (Figure 2, Supplementary Figure 2).

Besides, the crude rates of prevalence, incidence, and YLDs in women under 20 years of age, and the crude rates of mortality, DALYs, and YLLs in women under 40 years of age in 2019 were similar with those in 1990. Importantly, the OC burden in women over 40was increased to a large extent. The crude rates (per 100,000 population) of prevalence, incidence, and YLDs peaked between 55 and 59 years of age [27.41 (95% CI: 18.83, 35.45)], between 70 and 74 years of age [8.50 (95% CI: 5.48, 10.73)], and between 65 and 69 years of age [4.08 (95% CI: 2.33, 5.96)], respectively, while DALYs and YLLs both peaked between 65 and 69 years of age [DALYs rate: 158.08 (95% CI: 103.39, 203,19); YLLs rate: 154.00 (95% CI: 101.07, 198.70)]. In addition, the crude rates of mortality increased with age. The crude rates of mortality in OC patients over 95 years of age reached as high as 13.57 (95% CI: 9.67, 17.00) (Figure 2, Supplementary Figure 2). Thus, the OC burden in postmenopausal women and older women has increased a lot from 1990 to 2019 in China, while the OC burden in younger women remained relatively unchanged.

**Figure 3:**
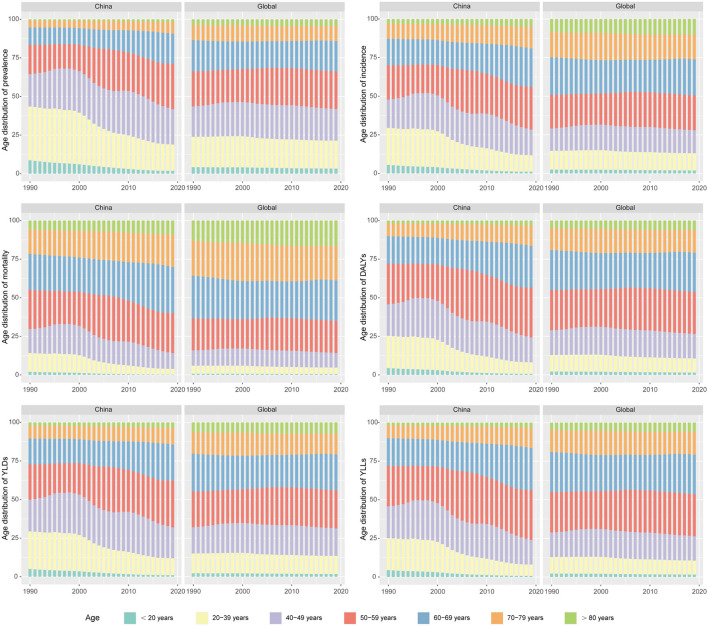
The trends in age distribution of ovarian cancer burden in China and the global level from 1990 to 2019.

Next, we compared the trends in age distribution of OC burden in China and the global level. Consistent with former results, age distribution of OC burden in the global level remained relatively stable from 1990 to 2019. In China, age distribution of OC burden exhibited a dynamic pattern. Over the past three decades, proportion of OC prevalence, incidence, mortality and DALYs in women under 40 years of age have apparently decreased year by year, while proportion of women over 50 years old showed a trend of increase year by year. As for women aged 40 to 49, their share of OC burden in all ages fluctuated over time. Age distribution of OC YLDs, and YLLs number showed similar trends (Figure 3).

### 3.3. Joinpoint regression analysis

**Figure 4:**
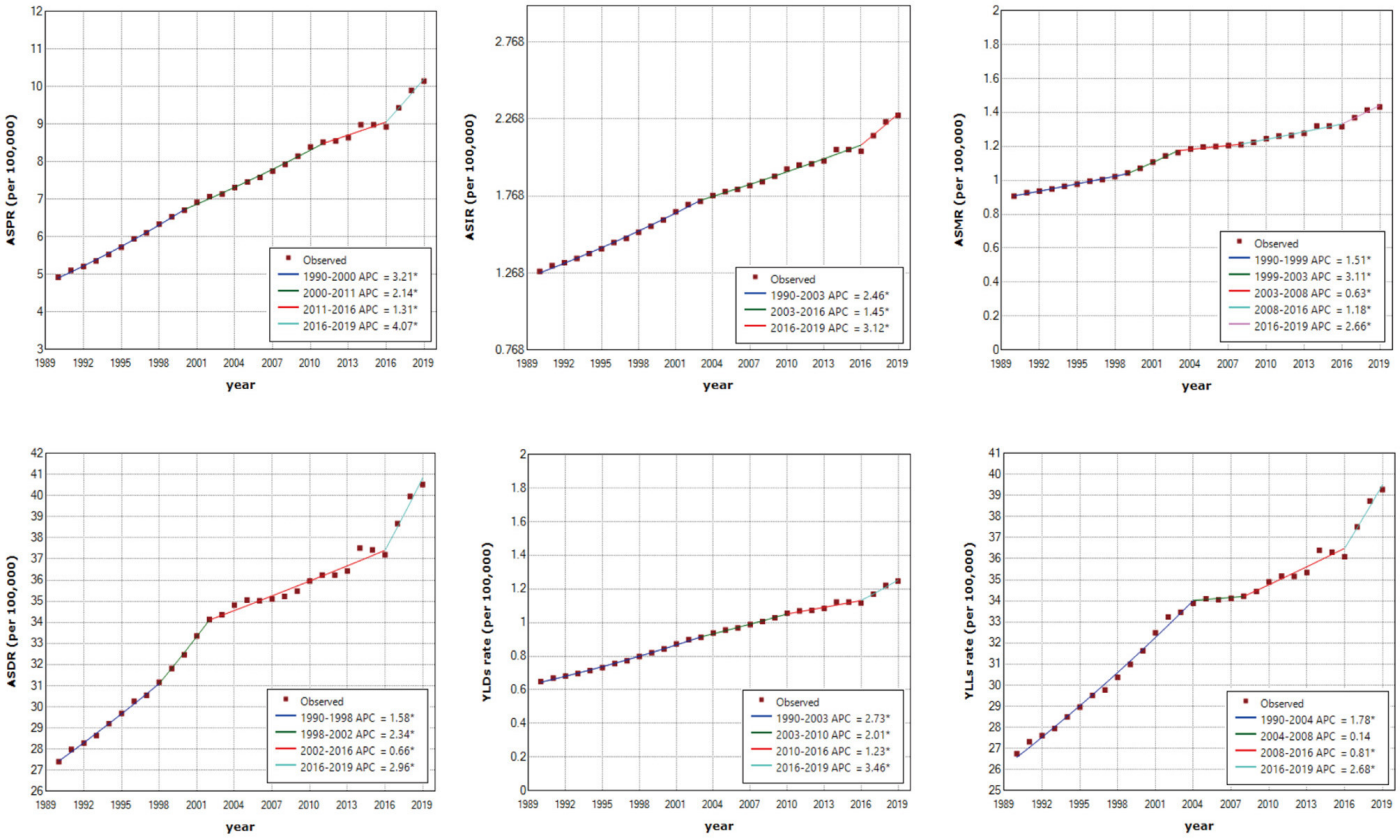
Joinpoint regression analysis of age-standardized prevalence rate (ASPR), age-standardized incidence rate (ASIR), age-standardized mortality rate (ASMR), age-standardized disability-adjusted life-years rate (ASDR), age-standardized years lived with disability rate (YLDs rate) and age-standardized years of life lost rate (YLLs rate) in China from 1990 to 2019. An asterisk indicates that the annual percentage change is statistically significantly different from zero at the α = 0.05 level.

Joinpoint regression analysis shows that the ASRs of OC burden indicators in China were all on the rise (Figure 4), while the OC burden in the global level was relatively stable (Supplementary Figure 3). Over three decades, the AAPCs of ASPR, ASIR, ASMR, ASDR, ASRs of YLDs and YLLs of OC in China were 2.56 (95% CI: 2.37, 2.75), 2.08 (95% CI: 1.94, 2.22), 1.60 (95% CI: 1.42, 1.78), 1.38 (95% CI: 1.18, 1.59), 2.32 (95% CI: 2.16, 2.47), 1.38 (95% CI: 1.20, 1.55), respectively, while the AAPCs in the global level fluctuated around zero (Table 2). Although the growing trend of ASPR from 2011 to 2016, ASMR from 2001 to 2016, and ASR of YLLs from 2004 to 2008 in China have moderated, it is noting that the increase rates of all the OC burden indicators were significantly accelerated from 2016 to 2019 (Figure 4), with APCs of ASPR, ASIR, ASMR, ASDR, ASRs of YLDs and YLLs were 4.07 (95% CI: 2.71, 5.45), 3.13 (95% CI: 1.88, 4.38), 2.66 (95% CI: 1.65, 3.68), 2.96 (95% CI: 1.67, 4.28), 3.46 (95% CI: 2.41, 4.53), 2.68 (95% CI: 1.44, 3.93), respectively (Table 2).

**Table 2:** Joinpoint regression analysis: trends in age-standardized rates of incidence, prevalence, mortality, DALYs, YLDs, and YLLs (per 100,000 population) in China and Global level from 1990 to 2019.

**Measure**	**China**			**Global**		
	**Period**	**APC (95% CI)**	**AAPC (95% CI)**	**Period**	**APC (95% CI)**	**AAPC (95% CI)**
ASPR	1990–2000	3.21 (3.02, 3.40)^*^	2.56 (2.37, 2.75)^*^	1990–2000	0.70 (0.59, 0.82)^*^	0.46 (0.38, 0.55)^*^
	2000–2011	2.14 (1.99, 2.28)^*^		2000–2015	0.16 (0.10, 0.22)^*^	
	2011–2016	1.31 (0.66, 1.97)^*^		2015–2019	1.01 (0.48, 1.54)^*^	
	2016–2019	4.07 (2.71, 5.45)^*^				
ASIR	1990–2003	2.46 (2.33, 2.59)^*^	2.08 (1.94, 2.22)^*^	1990–2001	0.31 (0.20, 0.41)^*^	0.13 (0.06, 0.20)^*^
	2003–2016	1.45 (1.35, 1.56)^*^		2001–2013	−0.19 (−0.27, −0.11)^*^	
	2016–2019	3.13 (1.88, 4.38)^*^		2013–2019	0.42 (0.16, 0.68)^*^	
ASMR	1990–1999	1.51 (1.29, 1.73)^*^	1.60 (1.42, 1.78)^*^	1990–2002	0.03 (−0.05, 0.10)	−0.13 (−0.19, −0.07)^*^
	1999–2003	3.11 (2.25, 3.98)^*^		2002–2012	−0.46 (−0.56, −0.36)^*^	
	2003–2008	0.63 (0.21, 1.07)^*^		2012–2019	0.08 (−0.10, 0.26)	
	2008–2016	1.18 (0.99, 1.36)^*^				
	2016–2019	2.66 (1.65, 3.68)^*^				
ASDR	1990–1998	1.58 (1.27, 1.90)^*^	1.38 (1.18, 1.59)^*^	1990–2002	−0.02 (−0.08, 0.05)	−0.02 (−0.11, 0.07)
	1998–2002	2.34 (1.29, 3.39)^*^		2002–2011	−0.36 (−0.46, −0.26)^*^	
	2002–2016	0.66 (0.57, 0.76)^*^		2011–2016	0.14 (−0.18, 0.45)	
	2016–2019	2.96 (1.67, 4.28)^*^		2016–2019	0.73 (0.08, 1.38)^*^	
ASRs of YLDs	1990–2003	2.73 (2.61, 2.84)^*^	2.32 (2.16, 2.47)^*^	1990–2001	0.48 (0.39, 0.57)^*^	0.25 (0.19, 0.32)^*^
	2003–2010	2.01 (1.70, 2.32)^*^		2001–2015	−0.07 (−0.13, −0.00)^*^	
	2010–2016	1.23 (0.80, 1.65)^*^		2015–2019	0.78 (0.36, 1.18)^*^	
	2016–2019	3.46 (2.41, 4.53)^*^				
ASRs of YLLs	1990–2004	1.78 (1.67, 1.89)^*^	1.38 (1.20, 1.55)^*^	1990–2002	−0.03 (−0.10, 0.04)	−0.03 (−0.12, 0.06)
	2004–2008	0.14 (−0.65, 0.93)		2002–2011	−0.37 (−0.47, −0.27)^*^	
	2008–2016	0.81 (0.58, 1.04)^*^		2011–2016	0.14 (−0.18, 0.45)	
	2016–2019	2.68 (1.44, 3.93)^*^		2016–2019	0.72 (0.09, 1.36)^*^	

APC, annual percent change; AAPC, average annual percent change presented for full period; CI, confidence interval; ASPR, age-standardized prevalence rate; ASIR, age-standardized incidence rate; ASMR, age-standardized mortality rate; ASDR, age-standardized disability-adjusted life-years rate; YLDs, years lived with disability; YLLs, years of life lost; ASRs, age-standardized rates; 95% CI, 95% confidence interval;

* p < 0.05.

### 3.4. Age-period-cohort analysis for OC incidence and mortality rates in China

**Figure 5:**
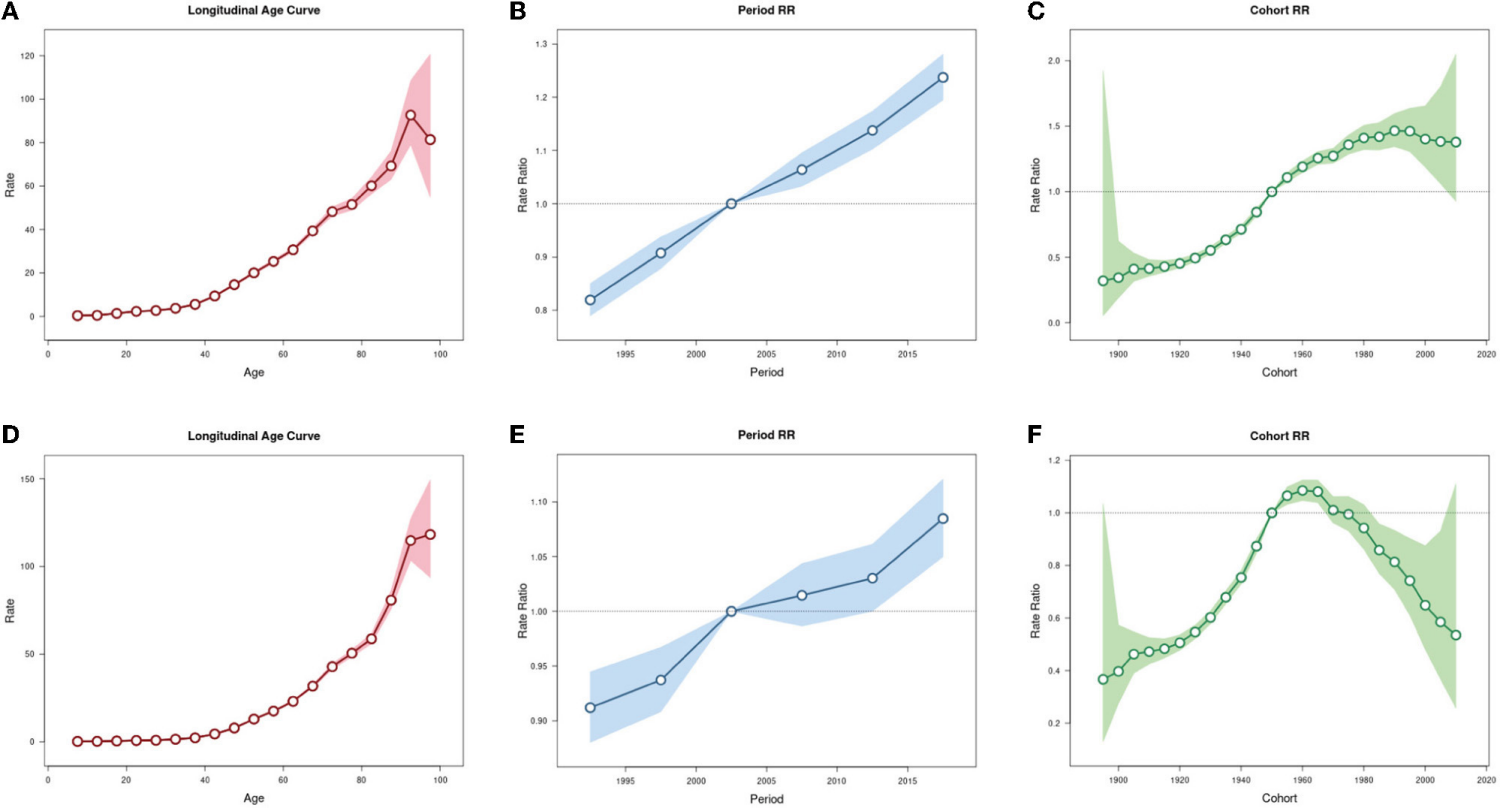
Age-period-cohort analysis for incidence and mortality rates of ovarian cancer in China. **(A)** Age effect for incidence rate. **(B)** Period effect for incidence rate. **(C)** Cohort effect for incidence rate. **(D)** Age effect for mortality rate. **(E)** Period effect for mortality rate. **(F)** Cohort effect for mortality rate.

The Net Drift (%) of OC incidence and mortality rates (per 100,000 population) in China are respectively 1.61 (95% CI: 1.43, 1.79) and 0.67 (95% CI: 0.50, 0.84), indicating that OC incidence and mortality rates in China were on the rise. The results shows that OC incidence rate of those younger than 12.5 years old and the mortality rate of those younger than 42.5 years old showed overall decreasing trends, while the incidence rate of those over 12.5 years old and the mortality rate of those over 42.5 years old showed overall decreasing trends (Supplementary Table 2). When the period and cohort effects were controlled, the incidence and mortality rates of OC in China increased with age (Figures 5A, D). For the period effect, the period RR of both incidence rate and mortality rate of OC kept increasing from 1990 to 2019 (Figures 5B, E). As for cohort effect, we found that the RR of OC incidence rate kept increasing in the cohort born before 1990, and then was gradually reduced by year of birth in the cohort born after 1990. Similarly, the RR of OC mortality rate first exhibited an increasing trend by year of birth, and then decreased, with the highest RR appeared at population born in 1960 (Figures 5C, F).

### 3.5. Trends of associated risk factors of OC from 1990 to 2019

**Figure 6:**
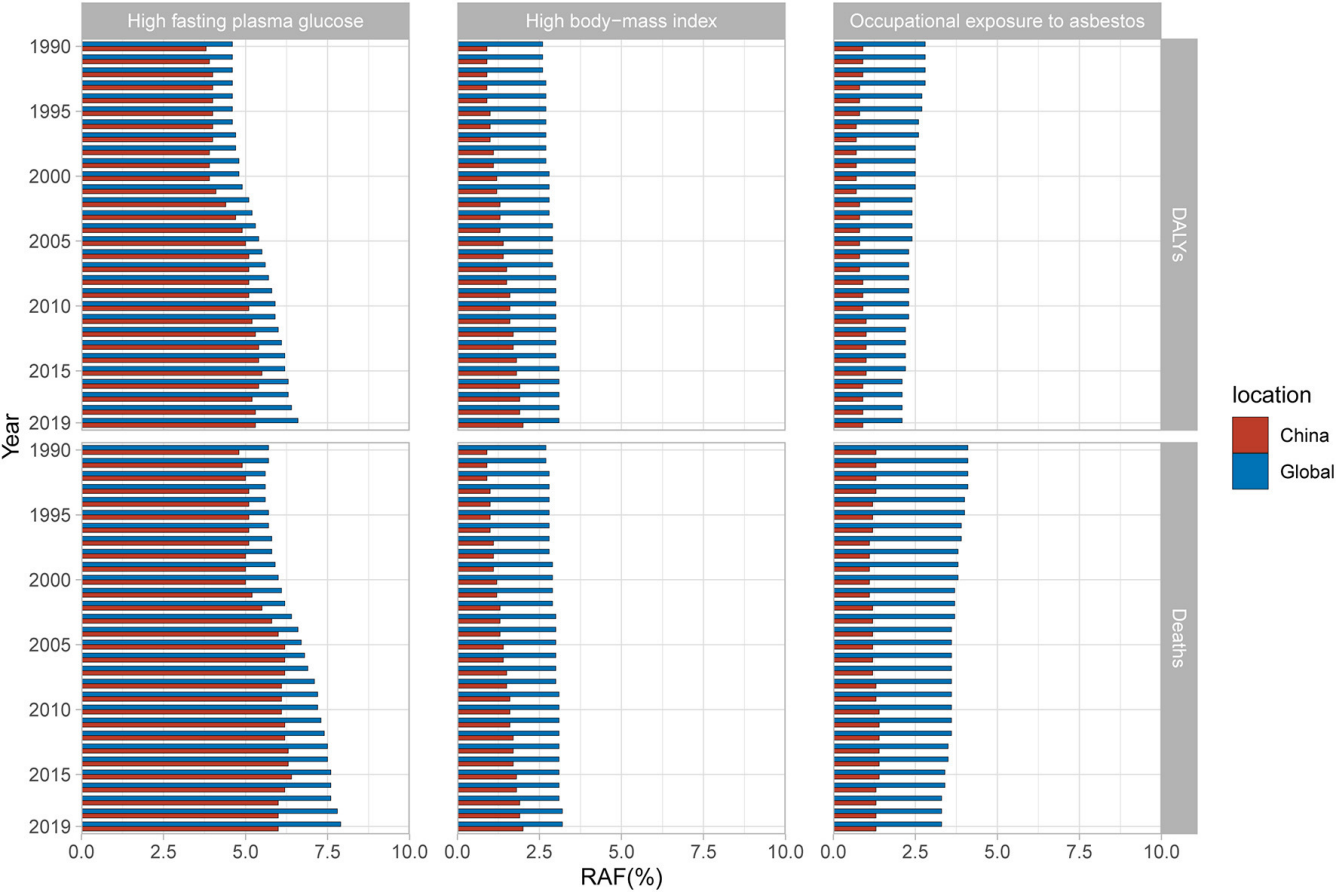
Population attributable proportion (PAF, %) of associated risk factors for ovarian cancer mortality and disability-adjusted life-years (DALYs) from 1990 to 2019.

GBD 2019 identified high fasting plasma glucose, occupational exposure to asbestos, and high body-mass index as three main risk factors for OC mortality and DALYs. In China, the largest contribution came from high fasting plasma glucose, with the PAF increasing rapidly from 2000 to 2005, and then fluctuating around 6.25%. The PAF of high fasting plasma glucose in the global level from 1990 to 2019 has risen year by year and was always higher than that of China. In addition, the PAF of high body-mass index has steadily increased year by year and has surpassed occupational exposure to asbestos to become the second largest contribution factor in China. Its growth rate is higher than that of the global level and is likely to catch up in the future. As for occupational exposure to asbestos, its contribution in China fluctuated from 1990 to 2019, while the global contribution has gradually decreased (Figure 6). Specific data is list in Supplementary Tables 3, 4.

### 3.6. The prediction of OC incidence and mortality rates from 2020 to 2030

**Figure 7:**
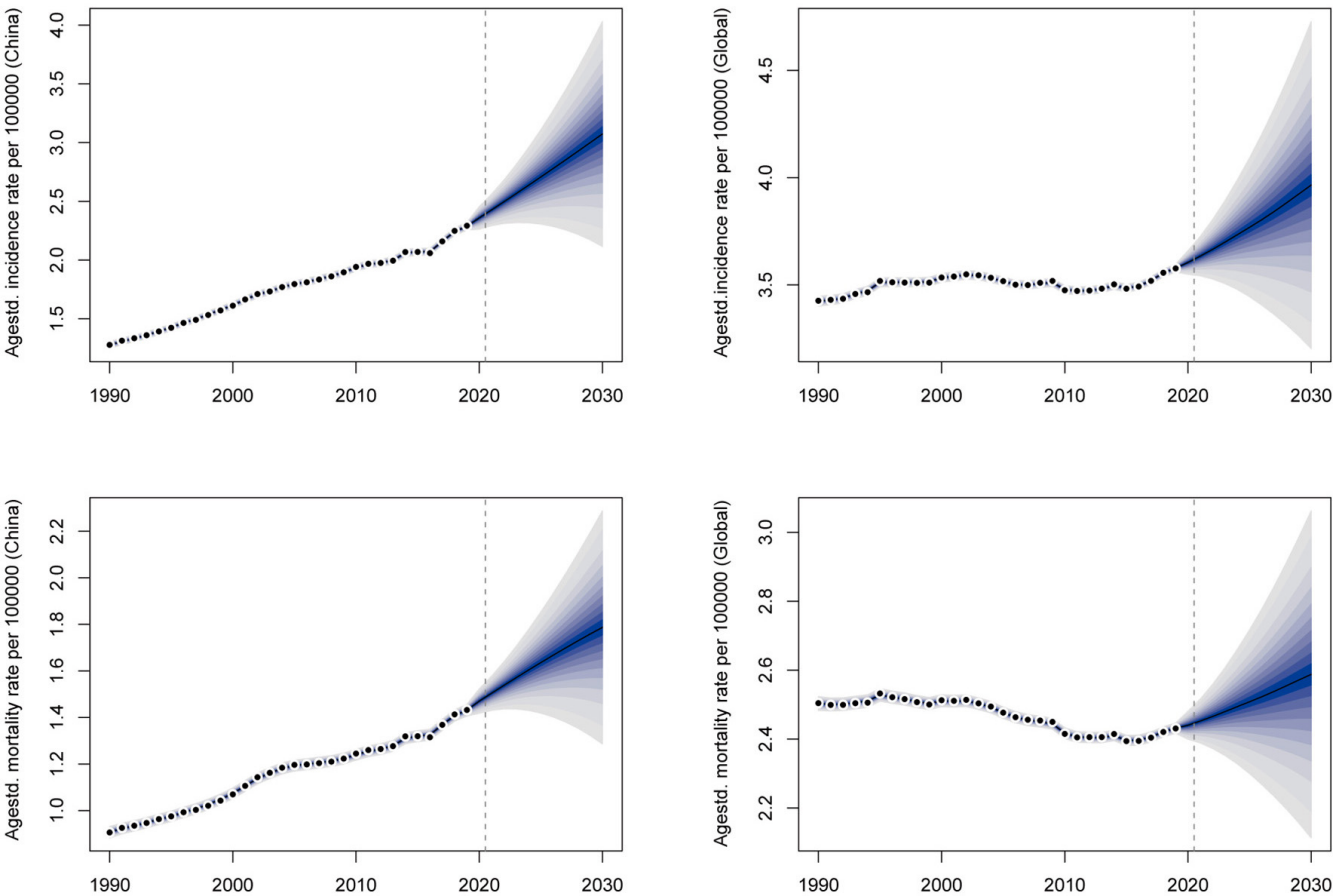
The prediction of ovarian cancer incidence and mortality rates for the next 10 years in China and the global level.

BAPC analysis results show that OC incidence and mortality rates will increase year by year in both China and the global level in the next decade. In 2030, the ASRs of OC incidence and mortality in China are expected to reach 2.96 (per 100,000 population) and 1.69 (per 100,000 population), respectively, with an increase of 29.26% and 18.18% compared with 2019. The number of incidence and mortality of OC in China will reach 72,700 and 46,100, respectively, increasing by 59.90% and 58.66% compared to 2019. At the same time, the ASRs of OC incidence and mortality in the global level are expected to reach 3.94 and 2.54 (per 100,000 population), respectively, with an increase of 10.06% and 4.53% compared to 2019. As for the global level, the numbers of OC incidence and mortality are expected to reach 410,000 and 274,200, with an increase of 39.28% and 38.24% by 2019, respectively (Figure 7). Annual data for OC incidence and mortality rates from 2019 to 2030 are shown in Supplementary Table 5. Above all, the burden of OC in China will become more and more serious in the next decade if there is no intervention, and the growth rate of which is significantly higher than the global level.

## 4. Discussion

Based on GBD 2019 data, we analyzed and compared the trend of OC burden in China and the global level from 1990 to 2030. In this study, we applied the joinpoint regression method to analyze OC burden trend in China for the first time, and firstly analyzed the changing trend of attributable risk factors for OC. The findings in our work will provide more reference for developing prevention and management measures for OC in China.

From 1990 to 2019, the numbers and crude rates of OC prevalence, incidence, mortality, DALYs, YLDs, YLLs have increased significantly in China. Although the overall global burden was also on the rise during the same period, the growth rate of OC burden in China was much higher than that in the global level, and the ASRs of OC burden indicators in the global level leveled off from 1990 to 2019. This is consistent with the results of a previous study (4), which reported that China has surpassed the United States to rank the first in terms of OC prevalence, mortality and DALYs numbers in the world. Over the past 30 years, the change trend of OC burden varies significantly across different sociodemographic index (SDI) regions. It's reported that OC prevalence, mortality and DALYs rates in high SDI regions like Western Europe, North America and Australia had a negative change, while those in low-middle SDI regions such as Asia, Latin America and Africa exhibited an increased change (4). The possible reason is that policy makers in high SDI regions pay more attention to the construction of advanced health care and environment, while in low-middle SDI regions, more attention is being paid to infrastructure improvement. The fact that the ASRs of these OC burden indicators were all less than the crude rates indicates that the significant increase of OC burden in China is partly owing to population growth and aging. In addition, the apparent increase in prevalence rate may be the result of the incidence rate being much greater than the mortality rate. In terms of mortality, our results are inconsistent with another previous study (3). This is possibly due to the result of GBD 2019 data algorithm optimization and update, which proved the necessity of our study.

On the whole, the burden of OC in women younger than 20 years old showed a downward trend, while those in women older than 40 years old increased significantly in China from 1990 to 2019. Hormone levels plummet in women after menopause, which is associated with an increased burden of OC (12). In general, OC patients before the age of 40 are mostly found in stage I and have a good prognosis, while those after the age of 40 are usually found at above stage II, and the prognosis is poor (13). In addition, the older individuals have deteriorated physical functions and are susceptible to a variety of underlying diseases (14). These may be the vital reasons for the high burden of OC in postmenopausal women and older women. From the perspective of age distribution, the proportion of OC burden in women under 40 years old showed a downward trend, while the proportion of women over 50 years old showed a significant upward trend from 1990 to 2019. And the proportion of women aged 40 to 49 showed a fluctuating trend. These results suggest that the younger trend of OC burden in China is not obvious. On the contrary, with the aging of the population, reducing OC burden in postmenopausal women and older women should become the focus of OC management and strategies in China.

It is worth noting that the OC burden in China had a slowing trend from 2014 to 2016, but after 2016, the growth trend increased significantly and even exceeded the previous maximum growth rate. Joinpoint regression analysis showed that the ASRs of various indicators of OC burden in China were also on the rise, and their AAPCs were about 1.38–2.56. In contrast, all indicators in the global level remained stable, and the AAPCs are close to zero. In China, there was a slowdown in the prevalence rate from 2011 to 2016 and the mortality rate from 2001 to 2016, but from 2016 to 2019 the growth rate of various indicators returned to the previous level or even became faster than ever before. This may be the result of increase in patient visit rate and OC detection rate due to the popularization of health education, medical insurance and advanced medical equipment. As for the global level, the relatively stable pattern of OC burden may be the result of the opposite trend between high SDI regions and low-middle SDI regions. BAPC projection of OC burden from 2019 to 2030 shows that OC incidence and mortality rates will keep on increasing in both China and the global level. But the burden in China will grow much faster than the global level. Therefore, the current situation of OC burden in China is severe, and it is urgent to find out the causes and take effective intervention measures.

We further analyzed the effects of age, period and cohort factors on OC incidence and mortality rates in China using the age-period-cohort method. From 1990 to 2019, the Net Drifts of OC incidence and mortality rates are both above zero, which verifies the grim situation of rapid growth of OC burden in China again. As expected, the age effect on OC incidence and mortality rates continuously increased with age. Similarly, the period effect on OC incidence rate showed an increasing trend during the whole period, indicating that the period effect was an important factor in the increasing trend of OC burden. Over the past 30 years, the economy, medical facility and life quality in China have improved a lot. At the same time, the pace of life has become faster, the mental pressure has gradually increased, and the lifestyle has changed fundamentally. In terms of eating habits, people used to eat enough vegetables rich in dietary fiber and vitamins. In recent years, obesity, diabetes, and sedentary behavior have become more prevalent. It's reported that dietary fiber (15) and vitamin intake (16) were inversely associated with OC risk, while obesity (17), diabetes (18), and sedentary behavior (19) are high risk factors for OC. In addition, studies have shown that duration of pregnancy (20) and breastfeeding (21) are negatively correlated with the risk of OC, so the fertility policy in China started in the 1980s may be one of the reasons for the increased risk of OC, but this remains to be verified. The cohort effect on OC incidence rate showed an increase trend in the cohort born before1990 and then exhibited a decrease trend in the cohort born after 1990. Similarly, the cohort effect on OC mortality rate showed an increase trend in the cohort born before 1960 and then exhibited a decrease trend in the cohort born after 1960. Poor environment, low socioeconomic levels and malnutrition in early childhood can profoundly and adversely affect health status, which may lead to higher OC risks in adulthood. The downward trend in the cohort effect may be a result of economic development, environmental improvement, universal access to health education, and improved medical conditions.

According to GBD 2019, high fasting plasma glucose is the major factor contributing the most to OC burden in China and the global level. The PAF of high fasting plasma glucose remains stable in recent years. High level of glucose provides sufficient energy for rapidly proliferation in cancer cells and promotes cancer development by activating insulin-like growth factor-1 receptor signaling pathways, altering programmed death receptor pathways, and modulating immune detection (22–25). It has been shown that diabetes is positively associated with OC mortality, and that hypoglycemic drugs such as metformin reduce the risk of OC in women (18, 26). A high body-mass index indicates obesity which could alter hormone levels and ovulation function and has been proved a major risk factor for OC (17). In China, the PAF of high body-mass index increased year by year with a faster growth rate than the global level from 1990 to 2019, and has surpassed occupational exposure to asbestos as the second most common attributing factor of OC. Therefore, it is especially necessary to promote healthy diet and strengthen physical exercise to prevent the occurrence of diabetes and obesity. Asbestos has been proved to cause OC by the International Agency for Research on Cancer. It's reported that up to twice the amount of asbestos fibers in the ovarian tissue of women with exposed family members compared to women without exposure (27). The asbestos fibers (without being able to be excreted) in the ovarian tissues lead to persistent local inflammation at the origin of tissue lesions and genetic and epigenetic alterations, which may promote ovarian tumorigenesis (28, 29). Asbestos has been classified as a Group 1 carcinogen by the World Health Organization since 1987 and has been banned to use in more than 60 countries (30). Thus, the PAF of occupational exposure to asbestos in the global level has decreased gradually, and is expected to be lower than high body-mass index. However, China has yet to completely prohibit the use of asbestos. As a result, occupational exposure to asbestos has become another major risk factor for OC and its PAF has remained relatively stable over the past 30 years. Finding alternative materials and using protective equipment to avoid direct exposure to asbestos may be beneficial to reduce the OC burden in China.

In recent years, China has made many efforts to control OC burden. Since 70% of OC patients are found in advanced stages due to its insidious onset, early detection and prevention are vital measures. Tumor suppressor genes BRCA1/2 play a role in maintaining genome stability by repairing double-strand DNA breaks. The pathogenic mutation in BRCA1/2 damages the function of BRCA1/2, leading to an increased risk of OC (31). Currently, women with a family history of OC in China are encouraged to be screened for the BRCA1/2 mutation to detect OC and start target treatment earlier. Some experts recommend the combination of serum CA125 (an epithelial carcinoma marker) and transvaginal ultrasound as an early screening method for OC in general women, but its effectiveness needs to be further demonstrated. To further promote quality control of clinical treatment, China has issued the latest quality control index for standardized diagnosis and treatment of primary ovarian cancer in 2022(32). We believe that the burden of OC in China will develop in a better direction in the future.

There are still some limitations in our study. Although GBD 2019 employs various adjusted methods to reduce data bias and the reliability has been confirmed by previous studies, these data are not direct measurements after all, which may lead to the inevitable bias (33, 34). Secondly, there are at least 50 risk factors of OC that have been reported so far (35), but the types of risk factors in GBD 2019 are limited and the analysis is not comprehensive enough. Thirdly, obvious differences in geographical environment, economic level and lifestyle are existing among provinces and regions in China. However, the corresponding data available for assessment is of lack so that this study did not specifically analyze the spatial distribution of OC burden in China. In the future, more efforts should be made to create and supplement OC burden data for provinces in China for deeper analysis. In addition, the COVID-19 outbreak has made a huge impact on society (36, 37), and trends in OC burden may change to some extent, but our projections do not take this into account.

In conclusion, the burden of OC in China has shown an obvious upward trend in the past 30 years, and the increase rate accelerated significantly in recent 5 years. In the next decade, OC burden in China will continue to rise with a higher rate than the global level. We found that the OC burden in people under 20 years of age is slowing down, while the OC burden in people over 40 years of age is getting more severe, especially in postmenopausal women and older women. Thus, these people would be the focus of efforts to OC management and prevention measures. Popularizing screening methods for OC, optimizing the quality of clinical diagnosis and treatment, promoting healthy lifestyle and reducing exposure to carcinogens will help reduce the future burden of OC in China.

## Data availability statement

The original contributions presented in the study are included in the article/Supplementary material, further inquiries can be directed to the corresponding author.

## Author contributions

Study concept and design: LH and YW. Data collection and quality control: HW and JP. Data analysis, construction of figures and tables, manuscript draft, and results interpretation: YW. Critical revision of the manuscript for important intellectual content: LH, ZW, and ZZ. All authors contributed to the article and approved the submitted version.
