# Supplementary material for: Burden of ovarian cancer in China from 1990 to 2030: A systematic analysis and comparison with the global level
Source: Front Public Health. 2023 Feb 13;11:1136596. doi: 10.3389/fpubh.2023.1136596 (PMC9969192; doi:10.3389/fpubh.2023.1136596)
Supplement: Supplementary file 1 [file Data_Sheet_1.docx]

Supplementary Material

Burden of ovarian cancer in China from 1990 to 2030: a systematic analysis and comparison with the global level

Ying Wang^1^, Zhi Wang^1^, Zihui Zhang^1^, Haoyu Wang^1^, Jiaxin Peng^1^, Li Hong^1*^

*** Correspondence:** Li Hong: dr_hongli@whu.edu.cn

# Supplementary Figures and Tables

## Supplementary Figures


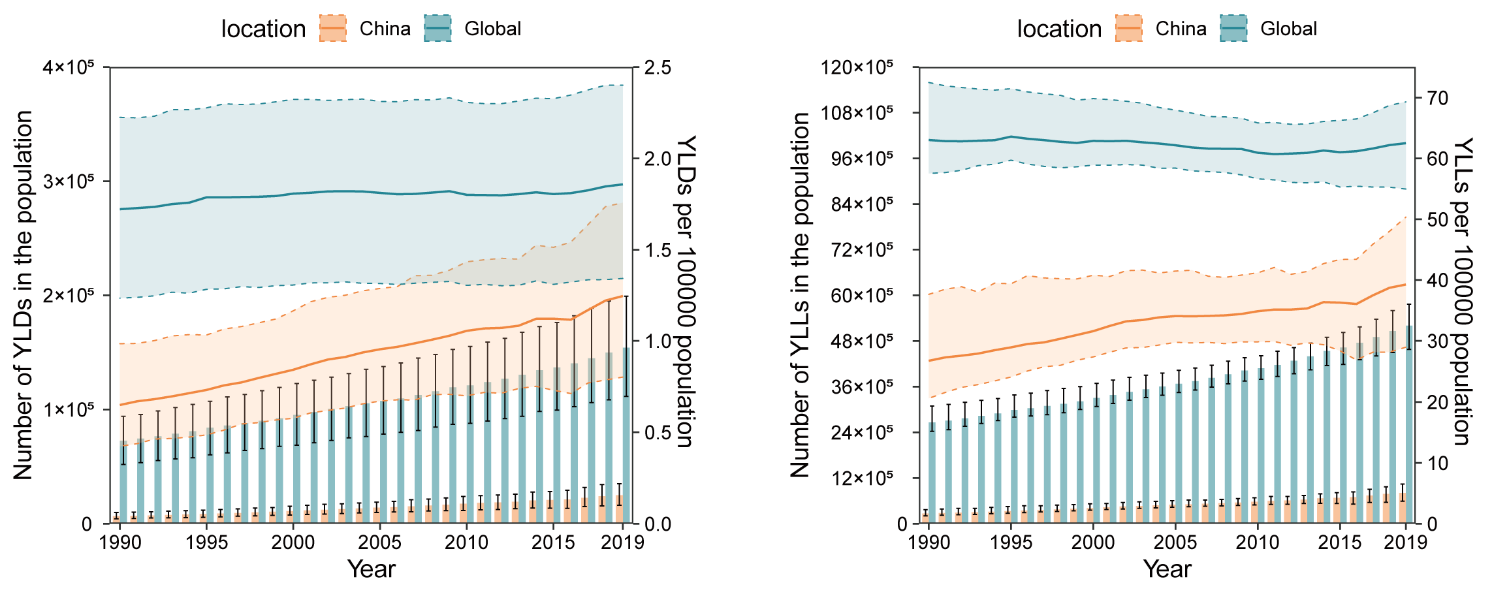


**Supplementary Figure 1** The numbers and age-standardized rates (per 100,000 population) of ovarian cancer years lived with disability (YLDs) and years of life lost (YLLs) from 1990 to 2019 in China and the global level. The bar chart represents numbers and the broken line chart represents age-standardized rates.


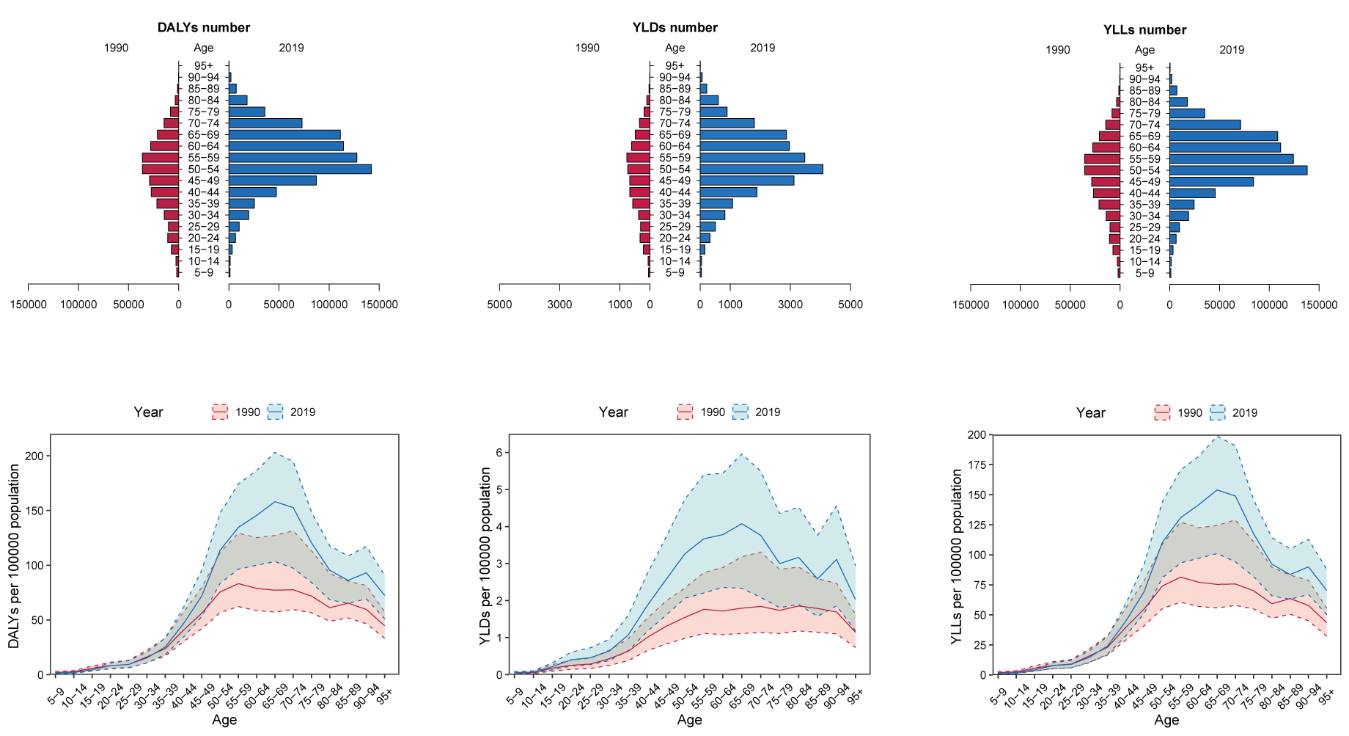


**Supplementary Figure 2** The numbers and crude rates (per 100,000 population) of ovarian cancer disability-adjusted life-years (DALYs), years lived with disability (YLDs) and years of life lost (YLLs) in 2019 compared with 1990 in China.


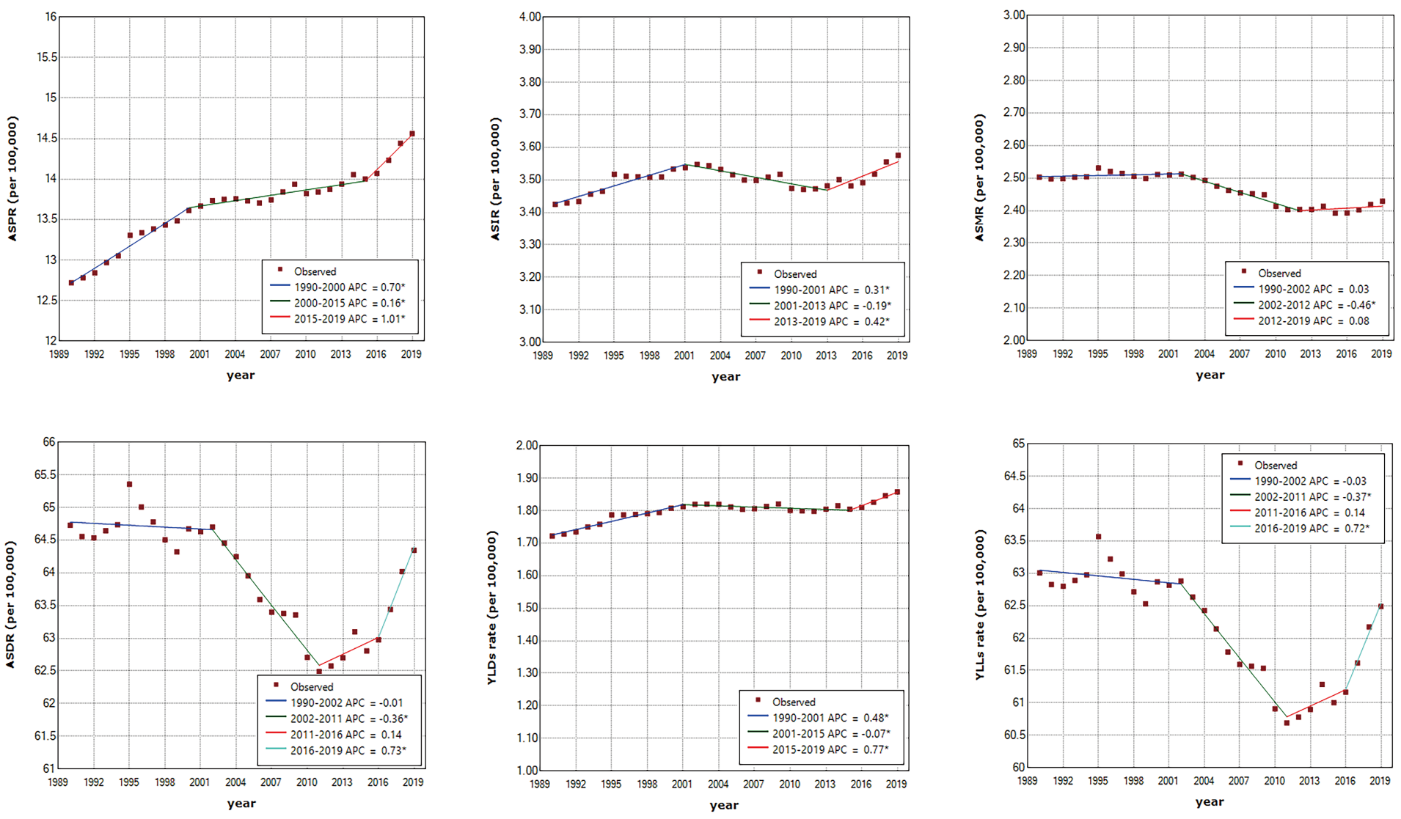


**Supplementary Figure 3** Joinpoint regression analysis of age-standardized prevalence rate (ASPR), age-standardized incidence rate (ASIR), age-standardized mortality rate (ASMR), age-standardized disability-adjusted life-years rate (ASDR), age-standardized years lived with disability rate (YLDs rate) and age-standardized years of life lost rate (YLLs rate) in the global level from 1990 to 2019. An asterisk indicates that the annual percentage change is statistically significantly different from zero at the α = 0.05 level.

## Supplementary Tables

**Supplementary Table 1** All-age numbers and crude rates (per 100,000 population) of prevalence, incidence, mortality, DALYs, YLDs and YLLs in 1990 and 2019 for ovarian cancer in China.

| **Measure** | **Number (95% CI)** | |  | **Crude rate (95% CI)** | |
| --- | --- | --- | --- | --- | --- |
|  | **1990** | **2019** |  | **1990** | 2019 |
| **Prevalence** | 54169.47 (42352.33, 72737.28) | 196349.32 (145037.56, 250677.74) |  | 4.58 (3.58, 6.15) | 13.80 (10.20, 17.62) |
| **Incidence** | 12680.35 (9905.45, 17511.49) | 45481.66 (33112.84, 57375.68) |  | 1.07 (0.84, 1.48) | 3.20 (2.33, 4.03) |
| **Mortality** | 8035.39 (6182.11, 11673.21) | 29092.06 (20956.30, 36859.81) |  | 0.68 (0.52, 0.99) | 2.05 (1.47, 2.59) |
| **DALYs** | 275059.61 (212261.36, 377971.81) | 835055.57 (612556.81, 1063246.71) |  | 23.24 (17.93, 31.93) | 58.71 (43.07, 74.75) |
| **YLDs** | 6580.22 (4290.75, 9810.8) | 24989.02 (16136.98, 35066.84) |  | 0.56 (0.36, 0.83) | 1.76 (1.13, 2.47) |
| **YLLs** | 268479.39 (206346.81, 370584.10) | 810066.55 (591493.82, 1040797.01) |  | 22.68 (17.43, 31.31) | 56.95 (41.59, 73.17) |

DALYs, disability-adjusted life-years; YLDs, years lived with disability; YLLs, years of life lost; 95% CI: 95% confidence interval.

**Supplementary Table 2** Local drifts (% per year) based on age-period-cohort analysis by age.

| **Age** | **Local drift (95% CI)** | |
| --- | --- | --- |
|  | **Incidence** | **Mortality** |
| **7.5** | -0.21 (-1.45, 1.06) | -1.97 (-4.20, 0.30) |
| **12.5** | -0.08 (-0.90, 0.75) | -1.88 (-3.31, -0.43) |
| **17.5** | 0.17 (-0.35, 0.70) | -1.65 (-2.58, -0.71) |
| **22.5** | 0.53 (0.16, 0.90) | -1.27 (-1.90, -0.64) |
| **27.5** | 0.65 (0.36, 0.94) | -1.12 (-1.58, -0.65) |
| **32.5** | 0.74 (0.49, 1.00) | -0.91 (-1.28, -0.54) |
| **37.5** | 0.93 (0.70, 1.15) | -0.54 (-0.83, -0.24) |
| **42.5** | 1.15 (0.96, 1.34) | -0.11 (-0.33, 0.12) |
| **47.5** | 1.62 (1.44, 1.79) | 0.56 (0.38, 0.75) |
| **52.5** | 2.29 (2.11, 2.47) | 1.45 (1.28, 1.61) |
| **57.5** | 2.69 (2.51, 2.87) | 2.03 (1.87, 2.19) |
| **62.5** | 2.91 (2.72, 3.10) | 2.40 (2.25, 2.56) |
| **67.5** | 2.85 (2.64, 3.07) | 2.45 (2.28, 2.62) |
| **72.5** | 2.52 (2.26, 2.78) | 2.20 (2.01, 2.40) |
| **77.5** | 2.11 (1.78, 2.46) | 1.85 (1.61, 2.09) |
| **82.5** | 1.72 (1.23, 2.21) | 1.47 (1.13, 1.81) |
| **87.5** | 1.20 (0.39, 2.01) | 1.04 (0.51, 1.57) |
| **92.5** | 1.23 (-0.54, 3.03) | 1.09 (-0.02, 2.20 |
| **97.5** | 1.38 (-0.38, 6.85) | 1.27 (-1.76, 4.41) |

95% CI: 95% confidence interval.

**Supplementary Table 3** Population attributable proportion (RAF, %) of risk factors contribute to ovarian cancer mortality in all ages from 1990 to 2019.

| **Year** | **RAF (95% CI) of**  **high fasting plasma glucose** | | **RAF (95% CI) of**  **high body-mass index** | | **RAF (95% CI) of**  **occupational exposure to asbestos** | |
| --- | --- | --- | --- | --- | --- | --- |
|  | **China** | **Global** | **China** | **Global** | **China** | **Global** |
| **1990** | 4.80 (0.92, 11.60) | 5.69 (1.11, 13.50) | 0.90 (-0.02, 2.81) | 2.71 (-0.08, 6.34) | 1.33 (0.60, 2.56) | 4.14 (1.89, 6.70) |
| **1991** | 4.93 (0.94, 11.87) | 5.66 (1.11, 13.42) | 0.91 (-0.02, 2.85) | 2.73 (-0.08, 6.38) | 1.32 (0.60, 2.58) | 4.11 (1.88, 6.63) |
| **1992** | 5.02 (0.96, 12.07) | 5.64 (1.11, 13.33) | 0.93 (-0.02, 2.88) | 2.75 (-0.08, 6.40) | 1.30 (0.59, 2.57) | 4.07 (1.89, 6.56) |
| **1993** | 5.10 (0.97, 12.32) | 5.62 (1.11, 13.27) | 0.95 (-0.02, 2.93) | 2.78 (-0.08, 6.45) | 1.29 (0.60, 2.42) | 4.08 (1.89, 6.57) |
| **1994** | 5.13 (0.99, 12.41) | 5.63 (1.11, 13.27) | 0.98 (-0.02, 2.99) | 2.79 (-0.08, 6.49) | 1.23 (0.56, 2.37) | 4.03 (1.90, 6.44) |
| **1995** | 5.11 (1.00, 12.33) | 5.66 (1.12, 13.34) | 1.00 (-0.02, 3.05) | 2.81 (-0.08, 6.53) | 1.18 (0.54, 2.21) | 4.00 (1.88, 6.37) |
| **1996** | 5.10 (0.99, 12.29) | 5.72 (1.14, 13.48) | 1.03 (-0.02, 3.10) | 2.82 (-0.08, 6.49) | 1.15 (0.52, 2.13) | 3.94 (1.88, 6.29) |
| **1997** | 5.07 (0.99, 12.28) | 5.78 (1.15, 13.59) | 1.07 (-0.03, 3.18) | 2.83 (-0.08, 6.53) | 1.13 (0.54, 2.08) | 3.88 (1.85, 6.22) |
| **1998** | 5.02 (0.98, 12.22) | 5.85 (1.17, 13.77) | 1.11 (-0.03, 3.25) | 2.85 (-0.08, 6.55) | 1.10 (0.51, 2.03) | 3.84 (1.83, 6.13) |
| **1999** | 4.99 (0.97, 12.07) | 5.92 (1.18, 13.90) | 1.15 (-0.03, 3.34) | 2.87 (-0.08, 6.58) | 1.09 (0.51, 1.95) | 3.82 (1.83, 6.05) |
| **2000** | 5.03 (0.98, 12.15) | 5.98 (1.19, 14.09) | 1.19 (-0.03, 3.44) | 2.89 (-0.08, 6.62) | 1.11 (0.53, 1.99) | 3.77 (1.81, 5.97) |
| **2001** | 5.18 (1.01, 12.46) | 6.08 (1.21, 14.29) | 1.23 (-0.03, 3.53) | 2.90 (-0.08, 6.60) | 1.12 (0.51, 2.04) | 3.74 (1.79, 5.90) |
| **2002** | 5.45 (1.08, 13.02) | 6.23 (1.25, 14.62) | 1.27 (-0.03, 3.69) | 2.93 (-0.08, 6.66) | 1.15 (0.55, 2.06) | 3.72 (1.78, 5.91) |
| **2003** | 5.77 (1.15, 13.73) | 6.41 (1.29, 14.99) | 1.31 (-0.03, 3.80) | 2.95 (-0.09, 6.71) | 1.19 (0.56, 2.12) | 3.70 (1.78, 5.88) |
| **2004** | 6.02 (1.21, 14.26) | 6.57 (1.33, 15.36) | 1.35 (-0.03, 3.85) | 2.97 (-0.09, 6.76) | 1.20 (0.56, 2.13) | 3.64 (1.75, 5.76) |
| **2005** | 6.15 (1.23, 14.53) | 6.68 (1.35, 15.59) | 1.39 (-0.04, 3.96) | 2.99 (-0.09, 6.80) | 1.19 (0.56, 2.12) | 3.62 (1.71, 5.69) |
| **2006** | 6.18 (1.24, 14.54) | 6.81 (1.38, 15.85) | 1.43 (-0.04, 4.06) | 3.00 (-0.09, 6.82) | 1.20 (0.54, 2.03) | 3.61 (1.66, 5.64) |
| **2007** | 6.17 (1.24, 14.59) | 6.95 (1.41, 16.12) | 1.47 (-0.04, 4.13) | 3.02 (-0.09, 6.86) | 1.22 (0.56, 2.09) | 3.62 (1.68, 5.71) |
| **2008** | 6.14 (1.23, 14.46) | 7.08 (1.44, 16.37) | 1.51 (-0.04, 4.24) | 3.04 (-0.09, 6.93) | 1.28 (0.58, 2.17) | 3.65 (1.70, 5.74) |
| **2009** | 6.12 (1.21, 14.41) | 7.19 (1.46, 16.61) | 1.56 (-0.04, 4.31) | 3.06 (-0.09, 7.00) | 1.33 (0.57, 2.19) | 3.63 (1.65, 5.80) |
| **2010** | 6.13 (1.21, 14.43) | 7.24 (1.47, 16.72) | 1.59 (-0.05, 4.39) | 3.07 (-0.09, 6.97) | 1.39 (0.59, 2.32) | 3.58 (1.64, 5.74) |
| **2011** | 6.18 (1.22, 14.45) | 7.30 (1.49, 16.80) | 1.63 (-0.05, 4.47) | 3.07 (-0.09, 7.00) | 1.42 (0.60, 2.41) | 3.57 (1.62, 5.70) |
| **2012** | 6.23 (1.22, 14.61) | 7.38 (1.51, 16.98) | 1.67 (-0.05, 4.56) | 3.09 (-0.09, 7.04) | 1.43 (0.60, 2.45) | 3.55 (1.62, 5.70) |
| **2013** | 6.29 (1.23, 14.75) | 7.46 (1.53, 17.19) | 1.71 (-0.05, 4.64) | 3.10 (-0.09, 7.01) | 1.42 (0.57, 2.43) | 3.53 (1.60, 5.66) |
| **2014** | 6.34 (1.23, 14.90) | 7.52 (1.54, 17.35) | 1.75 (-0.05, 4.70) | 3.11 (-0.09, 7.07) | 1.39 (0.56, 2.40) | 3.48 (1.58, 5.58) |
| **2015** | 6.35 (1.23, 14.93) | 7.57 (1.55, 17.44) | 1.78 (-0.06, 4.77) | 3.12 (-0.09, 7.09) | 1.36 (0.55, 2.31) | 3.44 (1.56, 5.53) |
| **2016** | 6.16 (1.19, 14.53) | 7.61 (1.56, 17.55) | 1.82 (-0.06, 4.86) | 3.14 (-0.09, 7.10) | 1.34 (0.54, 2.38) | 3.39 (1.55, 5.48) |
| **2017** | 5.95 (1.15, 14.21) | 7.65 (1.55, 17.67) | 1.86 (-0.06, 4.92) | 3.14 (-0.09, 7.12) | 1.33 (0.55, 2.26) | 3.32 (1.51, 5.30) |
| **2018** | 5.96 (1.16, 14.23) | 7.75 (1.57, 17.85) | 1.90 (-0.06, 5.01) | 3.15 (-0.09, 7.09) | 1.33 (0.53, 2.38) | 3.30 (1.51, 5.38) |
| **2019** | 6.05 (1.18, 14.37) | 7.94 (1.62, 18.28) | 1.96 (-0.06, 5.11) | 3.18 (-0.09, 7.12) | 1.34 (0.55, 2.31) | 3.30 (1.52, 5.40) |

95% CI: 95% confidence interval.

**Supplementary Table 4** Population attributable proportion (RAF, %) of risk factors contribute to ovarian cancer DALYs in all ages from 1990 to 2019.

| **Year** | **RAF (95% CI) of**  **high fasting plasma glucose** | | **RAF (95% CI) of**  **high body-mass index** | | **RAF (95% CI) of**  **occupational exposure to asbestos** | |
| --- | --- | --- | --- | --- | --- | --- |
|  | **China** | **Global** | **China** | **Global** | **China** | **Global** |
| **1990** | 3.78 (0.71, 9.37) | 4.62 (11.07, 0.90) | 0.86 (-0.02, 2.70) | 2.60 (-0.08, 6.15) | 0.89 (0.38, 1.84) | 2.82 (1.28, 4.55) |
| **1991** | 3.89 (0.74, 9.66) | 4.60 (11.04, 0.91) | 0.88 (-0.02, 2.74) | 2.62 (-0.08, 6.15) | 0.88 (0.39, 1.77) | 2.79 (1.26, 4.54) |
| **1992** | 3.96 (0.75, 9.85) | 4.58 (10.98, 0.90) | 0.90 (-0.02, 2.78) | 2.64 (-0.08, 6.20) | 0.86 (0.37, 1.75) | 2.76 (1.26, 4.49) |
| **1993** | 4.01 (0.77, 10.01) | 4.57 (10.97, 0.91) | 0.92 (-0.02, 2.83) | 2.67 (-0.08, 6.26) | 0.84 (0.38, 1.68) | 2.77 (1.26, 4.49) |
| **1994** | 4.02 (0.77, 9.85) | 4.58 (10.97, 0.91) | 0.94 (-0.02, 2.88) | 2.68 (-0.08, 6.29) | 0.80 (0.36, 1.58) | 2.73 (1.27, 4.42) |
| **1995** | 3.99 (0.77, 9.87) | 4.60 (11.03, 0.92) | 0.97 (-0.02, 2.95) | 2.70 (-0.08, 6.33) | 0.76 (0.34, 1.46) | 2.69 (1.24, 4.34) |
| **1996** | 3.98 (0.77, 9.78) | 4.64 (11.13, 0.93) | 1.00 (-0.02, 3.00) | 2.70 (-0.08, 6.27) | 0.74 (0.33, 1.40) | 2.64 (1.24, 4.23) |
| **1997** | 3.96 (0.76, 9.65) | 4.69 (11.24, 0.94) | 1.04 (-0.02, 3.09) | 2.72 (-0.08, 6.32) | 0.72 (0.34, 1.38) | 2.59 (1.23, 4.20) |
| **1998** | 3.91 (0.76, 9.50) | 4.74 (11.35, 0.95) | 1.08 (-0.03, 3.17) | 2.73 (-0.08, 6.34) | 0.70 (0.32, 1.31) | 2.55 (1.19, 4.07) |
| **1999** | 3.90 (0.75, 9.50) | 4.79 (11.46, 0.96) | 1.12 (-0.03, 3.28) | 2.75 (-0.08, 6.37) | 0.70 (0.32, 1.29) | 2.53 (1.18, 4.04) |
| **2000** | 3.94 (0.77, 9.61) | 4.84 (11.55, 0.97) | 1.16 (-0.03, 3.38) | 2.77 (-0.08, 6.41) | 0.71 (0.33, 1.30) | 2.49 (1.17, 3.98) |
| **2001** | 4.09 (0.80, 9.94) | 4.93 (11.72, 0.98) | 1.20 (-0.03, 3.47) | 2.79 (-0.08, 6.38) | 0.73 (0.33, 1.34) | 2.46 (1.16, 3.90) |
| **2002** | 4.36 (0.86, 10.50) | 5.06 (11.98, 1.01) | 1.25 (-0.03, 3.63) | 2.82 (-0.08, 6.46) | 0.76 (0.35, 1.40) | 2.44 (1.15, 3.90) |
| **2003** | 4.66 (0.92, 11.09) | 5.20 (12.33, 1.05) | 1.30 (-0.03, 3.76) | 2.85 (-0.08, 6.52) | 0.80 (0.37, 1.42) | 2.43 (1.14, 3.90) |
| **2004** | 4.90 (0.98, 11.63) | 5.33 (12.63, 1.08) | 1.34 (-0.03, 3.82) | 2.87 (-0.08, 6.57) | 0.81 (0.38, 1.45) | 2.38 (1.13, 3.81) |
| **2005** | 5.03 (1.01, 11.98) | 5.42 (12.82, 1.10) | 1.38 (-0.04, 3.94) | 2.89 (-0.08, 6.61) | 0.81 (0.39, 1.43) | 2.36 (1.11, 3.75) |
| **2006** | 5.09 (1.02, 12.08) | 5.52 (13.05, 1.12) | 1.42 (-0.04, 4.05) | 2.91 (-0.08, 6.64) | 0.82 (0.37, 1.40) | 2.34 (1.09, 3.73) |
| **2007** | 5.11 (1.02, 12.12) | 5.63 (13.27, 1.14) | 1.47 (-0.04, 4.14) | 2.93 (-0.08, 6.69) | 0.83 (0.38, 1.43) | 2.34 (1.08, 3.74) |
| **2008** | 5.10 (1.01, 12.11) | 5.74 (13.45, 1.17) | 1.52 (-0.04, 4.24) | 2.95 (-0.09, 6.74) | 0.87 (0.39, 1.50) | 2.34 (1.09, 3.75) |
| **2009** | 5.11 (1.01, 12.09) | 5.83 (13.62, 1.19) | 1.56 (-0.04, 4.32) | 2.97 (-0.09, 6.82) | 0.91 (0.39, 1.49) | 2.32 (1.07, 3.73) |
| **2010** | 5.14 (1.01, 12.17) | 5.88 (13.76, 1.19) | 1.60 (-0.05, 4.42) | 2.98 (-0.09, 6.78) | 0.94 (0.39, 1.59) | 2.28 (1.05, 3.64) |
| **2011** | 5.21 (1.02, 12.28) | 5.94 (13.89, 1.21) | 1.64 (-0.05, 4.51) | 3.00 (-0.09, 6.83) | 0.97 (0.40, 1.64) | 2.27 (1.03, 3.61) |
| **2012** | 5.29 (1.03, 12.52) | 6.03 (14.11, 1.23) | 1.69 (-0.05, 4.59) | 3.01 (-0.09, 6.88) | 0.98 (0.39, 1.69) | 2.25 (1.02, 3.59) |
| **2013** | 5.37 (1.04, 12.71) | 6.11 (14.29, 1.25) | 1.73 (-0.05, 4.68) | 3.03 (-0.09, 6.87) | 0.98 (0.37, 1.69) | 2.23 (1.00, 3.60) |
| **2014** | 5.45 (1.04, 12.89) | 6.19 (14.46, 1.27) | 1.77 (-0.05, 4.75) | 3.05 (-0.09, 6.92) | 0.97 (0.37, 1.70) | 2.20 (1.00, 3.56) |
| **2015** | 5.49 (1.05, 13.00) | 6.25 (14.57, 1.28) | 1.81 (-0.06, 4.83) | 3.06 (-0.09, 6.96) | 0.95 (0.37, 1.64) | 2.17 (0.99, 3.50) |
| **2016** | 5.37 (1.02, 12.77) | 6.30 (14.74, 1.28) | 1.85 (-0.06, 4.91) | 3.08 (-0.09, 6.96) | 0.94 (0.37, 1.68) | 2.15 (0.99, 3.49) |
| **2017** | 5.23 (1.00, 12.56) | 6.35 (14.83, 1.29) | 1.89 (-0.06, 4.99) | 3.09 (-0.09, 6.99) | 0.93 (0.37, 1.62) | 2.12 (0.96, 3.44) |
| **2018** | 5.25 (1.02, 12.54) | 6.45 (15.02, 1.29) | 1.94 (-0.06, 5.10) | 3.10 (-0.09, 6.96) | 0.94 (0.37, 1.70) | 2.11 (0.96, 3.42) |
| **2019** | 5.35 (1.03, 12.75) | 6.61 (15.28, 1.34) | 1.99 (-0.06, 5.20) | 3.13 (-0.09, 7.00) | 0.94 (0.38, 1.65) | 2.11 (0.96, 3.42) |

95% CI: 95% confidence interval.

**Supplementary Table 5** The predicted numbers and ASRs (per 100,000 population) of ovarian cancer incidence and mortality from 2020 to 2030.

| **Year** | **Incidence** | | | |  | **Mortality** | | | |
| --- | --- | --- | --- | --- | --- | --- | --- | --- | --- |
|  | **Mumber** | | **ASR** | |  | **Number** | | **ASR** | |
|  | **China** | **Global** | **China** | **Global** |  | **China** | **Global** | **China** | **Global** |
| **2020** | 47741 | 304624 | 2.36 | 3.60 |  | 30516 | 204960 | 1.47 | 2.44 |
| **2021** | 50115 | 313961 | 2.43 | 3.63 |  | 32079 | 211417 | 1.50 | 2.45 |
| **2022** | 52520 | 323264 | 2.50 | 3.66 |  | 33618 | 217685 | 1.54 | 2.46 |
| **2023** | 54898 | 332680 | 2.56 | 3.69 |  | 35096 | 223825 | 1.56 | 2.47 |
| **2024** | 57221 | 342452 | 2.62 | 3.72 |  | 36532 | 230127 | 1.58 | 2.47 |
| **2025** | 59514 | 352562 | 2.66 | 3.75 |  | 37964 | 236647 | 1.60 | 2.48 |
| **2026** | 61853 | 363062 | 2.71 | 3.78 |  | 39435 | 243438 | 1.61 | 2.48 |
| **2027** | 64299 | 374012 | 2.77 | 3.82 |  | 40960 | 250516 | 1.63 | 2.49 |
| **2028** | 66906 | 385544 | 2.82 | 3.86 |  | 42571 | 258004 | 1.65 | 2.51 |
| **2029** | 69704 | 397634 | 2.89 | 3.90 |  | 44297 | 265956 | 1.67 | 2.52 |
| **2030** | 72674 | 410065 | 2.96 | 3.94 |  | 46117 | 274206 | 1.69 | 2.54 |

ASR, age-standardized rate.
